# Beyond COX Inhibition: The Critical Role of the NF-κB Pathway in the Future of Anti-inflammatory Drugs

**DOI:** 10.34172/apb.46607

**Published:** 2026-05-19

**Authors:** Ian Jhemes Oliveira Sousa, Kerolayne de Melo Nogueira

**Affiliations:** ^1^Northeast Biotechnology Network, Focal Point on - Federal University of Piauí – UFPI, Teresina, Brazil; ^2^Federal University of Maranhão (UFMA), Bacabal, Brazil

## To Editor,

 Inflammation is a fundamental biological response essential for protecting the body against pathogens and tissue injury. However, when dysregulated, it contributes to the development of chronic inflammatory diseases, such as rheumatoid arthritis, inflammatory bowel disease, and psoriasis, substantially impairing quality of life and increasing the global healthcare burden.^1^ Amidst the multiplicity of molecular agents involved in inflammation, activated B-cell nuclear factor kappa-light-chain enhancer (NF-κB) emerges as a key transcription factor that orchestrates the expression of numerous genes involved in inflammatory processes.^2^

 Despite its critical role, NF-κB remains an underexplored therapeutic target, partly due to the complexity of its signaling pathways and concerns about potential side effects.^3^ Since the early 20th century, anti-inflammatory pharmacology has focused predominantly on inhibiting cyclooxygenase enzymes (COX-1 and COX-2), a strategy that, while effective, is inherently limited by adverse gastrointestinal and cardiovascular effects.^4,5^

 In contrast, NF-κB, despite regulating key inflammatory genes, including COX-2, remains a therapeutic target of remarkable potential that is still insufficiently explored in clinical pharmacology.^2,6^

 This letter argues that the slow translation of NF-κB biology into drug development represents a significant gap in pharmaceutical innovation. The development of nonsteroidal anti-inflammatory drugs (NSAIDs) has been a mainstay of modern medicine for over a century, with their efficacy largely attributed to the inhibition of cyclooxygenase enzymes (COX-1 and COX-2) and, consequently, to the reduction of prostaglandin synthesis.^7,8^

 This approach has revolutionized the management of pain and inflammation, but it is not without limitations. Chronic use of NSAIDs is associated with a significant burden of adverse effects, notably gastrointestinal (such as ulcers and bleeding) and cardiovascular (increased risk of thrombotic events), which restrict their long-term applicability and safety.^5^

 Therefore, the search for safer and more effective anti-inflammatory drugs remains an undeniable priority in pharmaceutical research.^9^ In parallel, nuclear factor kappa B (NF-κB) has emerged as a master regulator of the inflammatory response, orchestrating the expression of hundreds of pro-inflammatory genes, including cytokines, chemokines, adhesion molecules, and even COX-2 itself.^2,6^

 Dysregulated NF-κB activation is a common denominator in several chronic inflammatory diseases, such as rheumatoid arthritis, inflammatory bowel disease, and certain types of cancer.^10,11^ Despite its central role in the pathogenesis of inflammation, NF-κB has proven difficult to translate into safe and effective clinical therapies due to pathway ubiquity and on-target toxicities. The scientific literature widely recognizes the importance of NF-κB,^12^ but translating this knowledge into the development of a new class of anti-inflammatory drugs that act directly on its pathway has been remarkably slow, representing a significant gap in pharmaceutical innovation.

 This letter aims to highlight the unexplored potential of NF-κB as a therapeutic target for the development of next-generation anti-inflammatory drugs. We argue that a paradigm shift is needed, moving away from exclusive reliance on COX inhibition to embrace more comprehensive and targeted approaches that modulate the NF-κB pathway. We will explore the rationale for targeting NF-κB, the challenges inherent in its development as a pharmacological target, and the opportunities that arise from understanding its molecular complexity.

 Inhibition of COX enzymes acts in the final phase of the inflammatory cascade, blocking the production of prostaglandins. Although effective for acute symptoms, this approach does not address the myriad of inflammatory mediators that are activated upstream.^13^ NF-κB, on the other hand, is a transcription factor that controls the gene expression of a wide range of proteins involved in inflammation. NF-κB inhibition, therefore, has the potential to provide broader upstream control of the inflammatory response, suppressing not only prostaglandins, but also pro-inflammatory cytokines (such as TNF-α, IL-1β, IL-6), chemokines (such as MCP-1, IL-8) and adhesion molecules (such as ICAM-1, VCAM-1) that perpetuate chronic inflammation and tissue damage in various pathologies.^10,11^

 It is interesting to note that many classic NSAIDs, including acetylsalicylic acid (aspirin), have been shown to modulate NF-κB activity as a secondary effect, but were not designed to optimize this action.^14^ This suggests that some of the efficacy of NSAIDs may be inadvertently attributed to their ability to influence the NF-κB pathway. However, pharmaceutical research still seems to be stuck in the COX paradigm, as evidenced by the persistence in the development of selective COX-2 inhibitors (coxibs). Experience with coxibs, which were developed to reduce gastrointestinal effects, has revealed an increase in unfavorable cardiovascular outcomes, demonstrating that simple selectivity for COX-2 has not solved the safety problem and, in some cases, has created new challenges.^15^

 This reinforces the need to explore completely new targets and distinct mechanisms of action. The main barrier to the development of NF-κB inhibitors has been the challenge of achieving specificity while maintaining an acceptable safety profile. NF-κB is a ubiquitous signaling pathway, fundamental to innate immunity, cell survival, and proliferation, these fears have been legitimate concerns.^3^

 However, this view may be overly simplistic and ignores the inherent complexity of the NF-κB pathway itself. NF-κB is not a single entity, but rather a family of five proteins (RelA/p65, RelB, c-Rel, NF-κB1/p50, and NF-κB2/p52) that form different dimers, activated by canonical and non-canonical pathways.^16^ This complexity offers multiple opportunities for more subtle and selective modulation, rather than global inhibition. Basic research has already identified several promising intervention points, such as the inhibition of the IKK complex (IκB kinase), which is crucial for the canonical activation of NF-κB, or the prevention of the degradation of the IκBα inhibitor, which keeps NF-κB inactive in the cytoplasm.^17^

 Thus, we have argued that selective modulation of the canonical IKK–IκBα–RelA axis, while preserving host defense genes, represents a more translationally viable strategy than global inhibition of the NF-κB pathway in applications in the field of inflammatory diseases.

 In addition, modulation of specific isoforms or interference with specific protein-protein interactions within the pathway may allow the development of agents that selectively target the pathogenic aspects of NF-κB activation, minimizing unwanted side effects.^18^

 The reluctance to invest heavily in the clinical translation of NF-κB inhibitors represents a missed opportunity. For example, preclinical studies have shown that selective NF-κB inhibitors can suppress the production of inflammatory cytokines without completely compromising immune competence, indicating the potential for more refined therapeutic approaches that balance efficacy and safety.^19^

 The literature is full of natural and synthetic compounds that demonstrate potent anti-NF-κB activity in preclinical models, but few of these compounds advance to human trials.^20^ This scenario, although challenging, has shown signs of change in recent years, with a growing recognition of the potential of NF-κB and the development of new strategies for its selective modulation.^12^ The era of “brute force” COX inhibition needs to give way to a smarter, more targeted approach.

 The future of anti-inflammatory therapy does not lie in refining an already saturated mechanism of action, but in boldly exploring new horizons. Rather than representing a single pharmacological target, NF-κB constitutes a complex network of regulatory nodes that may be amenable to precision-based therapeutic modulation.

 The scientific community and the pharmaceutical industry must redirect their efforts to fill this gap, developing the next generation of anti-inflammatories that can finally offer high efficacy with a safety profile superior to that of traditional NSAIDs.^18^

 NF-κB inhibition represents a promising frontier in the development of anti-inflammatory therapies. Although challenges related to specificity and safety are real, a deeper understanding of the complexity of the NF-κB pathway paves the way for more selective and effective modulation strategies.^21^

 It is imperative that pharmaceutical research and industry invest in the clinical translation of NF-κB inhibitors, moving away from over-reliance on already established mechanisms of action. By doing so, we can unlock the full therapeutic potential of NF-κB and offer patients anti-inflammatory drugs that not only alleviate symptoms but also address the underlying causes of chronic inflammation with a significantly improved safety profile. Thus, the exploration of NF-κB as a therapeutic target deserves urgent attention and investment, marking a new frontier in inflammation research, poised to revolutionize the development of anti-inflammatory therapies.

## Competing Interests

 We declare that there is no conflict of interest.

## Ethical Approval

 Not applicable.
